# Whole-Genome Sequencing of *Lactobacillus shenzhenensis* Strain LY-73^T^

**DOI:** 10.1128/genomeA.00972-13

**Published:** 2013-11-21

**Authors:** Zhe Lin, Zhaoshan Liu, Rentao Yang, Yuanqiang Zou, Daiwei Wan, Jing Chen, Min Guo, Jiao Zhao, Chengxiang Fang, Ruifu Yang, Feng Liu

**Affiliations:** BGI-Shenzhen, Beishan Industrial Zone, Yantian District, Shenzhen, People's Republic of Chinaa; Shenzhen Key Laboratory of Bioenergy, BGI-Shenzhen, Shenzhen, People’s Republic of Chinab; School of Bioscience & Bioengineering, South China University of Technology, Guangzhou, People’s Republic of Chinac; State Key Laboratory of Pathogen and Biosecurity, Beijing Institute of Microbiology and Epidemiology, Beijing, People’s Republic of Chinad; China Center for Type Culture Collection (CCTCC), College of Life Sciences, Wuhan University, Wuhan, People’s Republic of Chinae

## Abstract

*Lactobacillus shenzhenensis* strain LY-73^T^ is a novel species which was first isolated from fermented goods. Here, we report the draft genome sequence of *Lactobacillus shenzhenensis* LY-73^T^.

## GENOME ANNOUNCEMENT

Lactic acid bacteria (LAB) are distributed in a variety of habitats rich in carbohydrate-containing substrates, including plants (1), naturally fermented products (2), and the gastrointestinal tracts of humans and animals (3). Most LAB play important roles in food fermentation and preservation (4, 5), and these bacteria are widely used for probiotics (6, 7). The genus *Lactobacillus* consists of 190 validated species and 6 species that have been published in the *International Journal of Systematic and Evolutionary Microbiology* (http://www.bacterio.cict.fr/) since the genus *Lactobacillus* was first proposed by Beijerinck (8).

*Lactobacillus shenzhenensis* strain LY-73^T^, isolated from fermented goods, is a novel species of the genus *Lactobacillus* (9). The draft whole-genome sequence of strain LY-73^T^ was determined by using a paired-end strategy with the platform Illumina HiSeq 2000. We generated a library with a median insert size of ~500 bp and then carried out paired-end sequencing (2 × 90-bp reads). After filtering low-quality reads, we finally obtained 2 × 2,777,778-bp quality filtered reads (totaling ~500 Mb); all of these reads provided ~153-fold coverage of the genome. The genome assembly was then achieved by SOAPdenovo 1.05 (10). All of the sequence reads were first generated into 330 contigs of >200 bp in size. We then ordered the contigs into 62 large scaffolds, with a size range of between 543 and 446,731 bp, and tried to fill in gaps in between the scaffolded contigs.

The draft genome sequence of LY-73^T^ was found to be 3,271,684 bp in length, with a G+C content of 56.44%. Open reading frames (ORFs) were predicted by Glimmer 3.0 (11). There were 3,188 possible ORFs found in the genome, and the average ORF length was 890 bp. Putative genes were then annotated by searching against the Swissprot, COG, and KEGG databases by BLAST (12), and additional annotation was done by use of the RAST (Rapid Annotations using Subsystems Technology) server (13). The entire genome contains double copies of 23S rRNA genes, single copies of 16S rRNA-encoding genes, and 43 predicted tRNA-encoding genes. Approximately 87.4% of nucleotides are predicted to encode proteins, and 72% (2,295) of the predicted proteins are annotatable with known proteins. Strain LY-73^T^ contains 22 putative two-component regulatory systems, which may play roles related to its ability to survive under complicated environmental conditions. Several genes related to bacteriocin were found, and genes encoding proteins related to carbohydrate metabolism were also annotated. There are many putative genes that play roles in defense mechanisms, such as ABC-type multidrug and antimicrobial peptide transport systems, suggesting the potential of this bacterium for probiotic use.

### Nucleotide sequence accession numbers.

This whole-genome shotgun project has been deposited at DDBJ/EMBL/GenBank under the accession number AVAA00000000. The version described in this paper is version AVAA01000000.
